# Effect of *Cinnamomum verum* leaf essential oil on virulence factors of *Candida* species and determination of the *in-vivo* toxicity with *Galleria mellonella* model

**DOI:** 10.1590/0074-02760200349

**Published:** 2020-09-25

**Authors:** Gayan Kanchana Wijesinghe, Flávia Camila Maia, Thaís Rossini de Oliveira, Simone N Busato de Feiria, Felipe Joia, Janaina Priscila Barbosa, Giovana Cláudia Boni, Janaina de Cássia Orlandi Sardi, Pedro Luiz Rosalen, José Francisco Höfling

**Affiliations:** 1 Universidade Estadual de Campinas, Faculdade de Odontologia de Piracicaba, Área de Microbiologia e Imunologia, Departamento de Diagnóstico Oral, Campinas SP, Brasil; 2 Universidade Estadual de Campinas, Faculdade de Odontologia de Piracicaba, Área de Farmacologia, Anestesiologia e Terapêutica, Departamento de Ciências Fisiológicas, Campinas, SP, Brasil

**Keywords:** Cinnamomum verum, essential oil, *Candida* spp., biofilms, anti-virulence, Galleria mellonella

## Abstract

**BACKGROUND:**

Essential oils (EO) extracted from *Cinnamomum verum* has been used as an antimicrobial agents for centuries. The effects of *C. verum* leaf oil against virulence of microorganisms is not well studied yet.

**OBJECTIVES:**

This study evaluates the effect of *C. verum* leaf oil against three virulence factors of *Candida albicans*, *C. tropicalis* and *C. dubliniensis* and its *in-vivo* toxicity.

**METHODS:**

Chemical composition of EO was determined using gas chromatography-mass spectrometry (GC-MS). Minimum inhibitory concentration (MIC) was determined using clinical and laboratory standards institute (CLSI) M27-A3 broth microdilution. Effect of EO on initial adhesion was quantified using XTT assay after allowing *Candida* cells to adhere to the polystyrene surface for 2 h. Biofilm formation of *Candida* in the presence of EO was quantified using XTT viability assay. Efficacy on reduction of germ tube formation was evaluated using standard protocol. Visualisation of biofilm formation and progression under the EO treatment were done using scanning electron microscope (SEM) and Time lapses microscope respectively. *In-vivo* toxicity of EO was determined using *Galleria mellonella* larvae. Chlorhexidine digluconate: positive control.

**RESULTS:**

Eugenol was the main compound of EO. MIC was 1.0 mg/mL. 50% reduction in initial adhesion was achieved by *C. albicans*, *C. tropicalis* and *C. dubliniensis* with 1.0, > 2.0 and 0.34 mg/mL respectively. 0.5 and 1.0 mg/mL significantly inhibit the germ tube formation. MBIC_50_ for forming biofilms were ≤ 0.35 mg/mL. 1.0 mg/mL prevent biofilm progression of *Candida*. SEM images exhibited cell wall damages, cellular shrinkages and decreased hyphal formation. No lethal effect was noted with *in-vivo* experiment model at any concentration tested.

**CONCLUSION:**

*C. verum* leaf oil acts against virulence factors of *Candida* and does not show any toxicity.

*Candida* spp. is the most common causative agent of opportunistic fungal infections, leading to a range of serious life-threatening invasive to non-life-threatening mucocutaneous diseases including skin and ear infections, genitourinary candidiasis, nosocomial pneumonias, medical device associated infections and Candidaemias.^1^ ^,^ ^2^ ^,^ ^3^ ^,^ ^4^

Some *Candida* species show commensalism and colonise the skin and mucosal surfaces of the human body. Severely ill or patients with compromised immunity are more prone to develop both superficial and life-threatening systemic *Candida* infections.^5^ Predisposing factors for the infections are: extremes of age, hormonal changes, nutritional deficiency, HIV incidence, frequent antimicrobial exposure, chemotherapeutic treatments, carbohydrate-rich (glucose) diets, use of prostheses and low immunity.^6^ ^,^ ^7^ ^,^ ^8^ ^,^ ^9^ as host factors. Based on the available data, the total occurrence of *Candida* infections is around 1.2-25 cases per 100 000 population or 0.19-2.5 per 1000 hospital admissions all over the world. 13% of these were reported with patients with underlying predisposing factor like compromised immunity etc.^10^ ^,^ ^11^

Among all *Candida* species, *Candida albicans* is the most frequent etiological agent in cases of fungal infections, being associated in up to 50% of cases of this disease.^12^ This species has the capacity to adapt and proliferate easily in the environment of the human body, in several places. This ability to survive in different tissues of the human body is related to its capacity for morphological transition between hyphal form and yeast form.^13^ According to Mayer et al., hyphae and yeast, are involved in the infection process, and the yeast, being smaller, is able to disseminate, while the hyphae invades the host tissue by penetrating the epithelium with the help of proteinases and phospholipases enzymes, escaping from phagocytic cells.^14^ ^,^ ^15^ ^,^ ^16^ ^,^ ^17^ Another factor associated with virulence of *Candida* spp. is the ability to form biofilms, promoting cell adhesion and formation of the extracellular matrix of the biofilm, as well as the synthesis of proteins that favor its further adhesion. The biofilm structure increases the resistance to antimicrobial agents, due to the difficulty of penetration into the extracellular matrix, and the action of the immune system, besides allowing the increase of the gene expression of mechanisms of resistance to antifungals as the efflux pumps.^18^

Same as many bacterial and fungal species, antimicrobial resistance is an emerging problem with the *Candida* spp. Centre for Disease Control and Prevention (CDC) reports that, about 7% and 1.6% of all systemic infection causing *Candida* isolates from year 2012-2016 demonstrated a resistance to the first line antifungal drug, fluconazole and to echinocandins.^19^ Hence, new therapeutic options are needed to effectively address the emerging issue of developing antimicrobial resistance, in addition to overcoming the toxicities and drug interactions that are associated with available antifungal agents.^20^ Plant derived natural therapeutics are becoming more popular among the vast majority of the modern world due to its low toxicity, easy accessibility, cost efficacy as well as the high therapeutic potency.^20^ In this aspect, *Cinnamomum verum*/ True Cinnamon, has been shown to be of great economic and pharmaceutical-medicinal interest.^21^

Extracted phytochemicals from various parts of the cinnamon tree (bark, leaves, fruits and flowers etc.) have been used as a culinary spice and medicinal plant since ancient times. It has also been used as an ingredient in many ancient Asian (especially in India, China and Sri Lanka) therapeutic preparations.^21^ In traditional folk medicine, cinnamon is used for the treatment of many disease conditions including eye inflammations, vaginitis, rheumatism, and neuralgia as well as wounds and toothaches apart from the use as an antimicrobial and anti-parasitic agent. Cinnamon is also useful for treatment of bronchitis, itching, and urinary tract and digestive tract related diseases.^22^

Although, many *in-vitro* microbiological studies with *C. verum* show antimicrobial activities related to many microbial species in the health care area, including those that exhibit multiple antimicrobial resistance such as *Acinetobacter* spp., *Bacillus* spp., *Enterobacter* spp., *Enterococcus faecalis*, *Escherichia coli*, *Mycobacterium tuberculosis*, *Klebsiella pneumoniae* and *Haemophilus influenzae* etc.,^22^ the anti-*Candida* activity, specially, effect on virulence of *Candida* spp. is still less comprehensive.

The present study aimed to evaluate the effect of *C. verum* leaf essential oils (EO) on the initial adhesion, germ tube formation and biofilm formation of *Candida* spp., and determine its toxicity *in-vivo* using *Galleria mellonella* larvae model.

## MATERIALS AND METHODS

*Fungal strains* - Three *Candida* type strains, *C. albicans* (ATCC MYA-2876), *C. tropicalis* (ATCC 750) and *C. dubliniensis* (ATCC MYA-646) obtained from Microbiology and Immunology Area, Faculty of Dentistry in Piracicaba, UNICAMP, Brazil, were used in this study. The standard strains of *Candida* spp. stocks were maintained in 80% grycerol in ultrafreezer at -80ºC. To reactivate stock organisms, they were subcultured in freshly prepared Saboraud Dextrose Agar (SDA, OXOID) culture medium, incubated aerobically at 37ºC for 24 h.

The standard inocula were prepared by adjusting the turbidity in accordance with McFarland scale (0.5), which was equivalent to the absorbance of 0.08-0.10 (600 nm) corresponding to 5 x 10^6^ colony forming unit (CFU)/mL.

*Essential oil* - The EO of *C. verum* leaves was purchased from Romik Lanka Marketing Services, Moratuwa, Sri Lanka (WCC/3569). The *C. verum* EO was diluted to 32 mg/mL in Tween 80 (0.05%) solution and Roswell Park Memorial Institute (RPMI) buffered with MOPS [3-(N-morpholino) propane sulfonic acid] followed by sonication, 1 cycle of 20 seconds.

*Chemical analysis of the EO* - The chemical analysis of *C. verum* EO was performed using HP-6890 (Agilent, USA) gas chromatograph coupled with HP-5975 (Agilent, USA) selective mass detector; HP-5MS Capillary Column (30 m x 0.25 mm x 0.25 µm); temperatures: injector (220ºC), detector (250ºC), column (60ºC), 3ºC/min, 240ºC; flow rate of carrier gas (highly dried He) of 1.0 mL/min^18^. 1.0 µL of oil was injected in split mode and the ionisation source was 70 eV. The relative abundance of the components were calculated using the following equation.^23^

Percentage abundance (%) = (Area of the component/total area of the chromatogram) × 100

The database for identifying the analytes was NIST-11 (National Institute of Standards and Technology) mass spectral database and NIST mass spectral search program (Version 2.0 g).

*Determination of minimum inhibitory concentration (MIC)* - MIC was determined using the clinical and laboratory standards institute (CLSI) M27-A3 broth microdilution method with modification.^24^ Briefly, 100 µL of the dilution of the EO in RPMI 1640 with MOPS was mixed with 100 µL of the prepared standard fungal cell suspensions (1 × 10^6^ cells/mL) in a 96 well sterile flat bottomed polystyrene microtiter plate followed by aerobic incubation at 37ºC for 24 h. After incubation, plates were visually observed for the presence and absence of growth (turbidity of the suspensions). Minimum concentration of the treatment without any turbidity was considered as MIC point.

Growth control: 100 µL of buffered RPMI 1640 instead of EO + standard cell suspension. Positive control: 120 mg/mL chlorhexidine digluconate was tested.

*Effect on initial adhesion* - Effect of *C. verum* leaf oil on *C. albicans* (ATCC MYA-2876), *C. dubliniensis* (ATCC MYA-646) and *C. tropicalis* (ATCC 750) adhesion to a sterile polystyrene surface was studied by using previously published methodology by Raut et al.^25^

Few colonies of 24 h old fresh *Candida* cultures on sabouraud dextrose agar (SDA) were inoculated into 50 mL of YPD broth in labeled sterile culture media bottle and incubated at 35-37ºC for 18-24 h in a shaking incubator at 30 rpm (Nova Instruments, Brazil).

After incubation, cells were harvested and washed twice with sterile phosphate buffered saline (PBS) and the inoculum was adjusted to 1.0 x 10^7^ cells/mL in RPMI 1640 medium after counting cells using Neubauer improved counting chamber.^24^

Ninety-six well sterile flat bottomed microtiter plates were seeded with these suspensions (100 µL/well) and allowed to adhere to polystyrene surface of plates for 2 h at 37ºC in a shaker incubator (75 rpm, Nova Instruments, Brazil), in the presence of different concentrations of *C. verum* leaf oil ranging from 2 mg/mL to 0.0039 mg/mL (100 µL/well). Wells without oil were kept as growth control. RPMI buffered with MOPS+0.05% Tween 80 was added to negative control wells. 120 mg/mL chlorhexidine digluconate in RPMI 1640 buffered with MOPS (Sigma-Aldrich, USA) was used as positive control.

After 2 h incubation, wells were washed with 200 µL sterile normal saline in order to remove non-adherent cells. Attached cells in each well were quantified using XTT metabolic assay. Briefly, 80 μL of XTT [2,3-Bis-(2-Methoxy-4-Nitro-5-Sulfophenyl)-2H-Tetrazolium-5-Carboxanilide, Sigma-Aldrich] and menadione (Sigma-Aldrich) solution (4 µL of menadione in 10 mL XTT) was added to each well and plates were then covered with a piece of aluminium paper to protect from light and incubated for 2 h at 37^º^C. Then, the absorbance of resulting solution was measured at 490 nm using microtiter plate reader /Versa MAX, molecular Devices, USA.^26^

Percentage reduction of adhered cells was calculated by comparing the mean XTT activity of the test groups and negative control group.^24^

*Effect on germ tube formation* - The formation of germ tubes from blastoconidia is the first step in the development of true hyphae and the ability to switch between morphological forms has been suggested as a potential virulence factor. Compared to the unicellular blastoconidial form, hyphae have an increased ability to adhere to and penetrate host tissues.

An experiment described by Hammer et al.^27^ was carried out to examine the effects of *C. verum* leaf oil on the morphological transition (germ tube/hyphal formation) from blastoconidia to hypha of *C. albicans* (ATCC MYA-2876) and *C. dubliniensis* (ATCC MYA-646).

Three concentrations (1/4 MIC, 1/2 MIC and MIC) of *C. verum* leaf oil and chlorhexidine digluconate were prepared in foetal bovine serum (FBS) (Sigma-Aldrich) in 1 mL volumes in sterile glass Bijou bottles at twice the desired final concentration as follows: 0.25, 0.5, 1.0 and 0 mg/mL.

Equal volumes (1 mL) of the prepared cell suspensions in yeast extract-peptone-dextrose (YPD) medium as explained previously were added to each *C. verum* leaf oil treatment and mixed thoroughly. All treatments were then incubated aerobically at 37ºC, without shaking, and were sampled at 0 h, 2 h, 4 h and 6 h after mixing thoroughly.

10 μL of prepared suspensions were loaded to Neubauer improved counting chamber at each time point and the number of germinated cells (Cells with germ tubes) in 500 *Candida* cells was counted and the percentage of germ tube formation was calculated by dividing the number of germinated cells by five.

*Effect on biofilm development in the presence of (EO)* - 100 μL of standard inoculum of each organism was inoculated to a 96-well sterile flat bottomed microplate plate, followed by aerobic incubation for 120 minutes under agitation (75 rpm at 37ºC)( Nova Instruments, Brazil). The plate was then washed once with sterile PBS and 100 μL of the each dilution of the essential oil was added separately to the treatment wells. 100 μL of RPMI 1640 buffered with MOPS was added to the growth control wells and buffered RPMI 1640 with 0.05% Tween 80 was added to negative control wells instead of oil.^28^ Chlorhexidine digluconate was used as positive control. The plate was then aerobically incubated for 24 h at 37ºC.

Biofilm biomass was quantified after 24 h incubation using XTT metabolic assay explained previously.

*Minimum toxic concentration for forming biofilms (MTC)* - MTC determines the minimum concentration of *C. verum* leaf oil requires to kill the forming biofilms of *Candida* spp. completely. The CFU assay was used to detect the MTC of the oil on forming biofilms. After the 24 h treatment with different concentrations of *C. verum* leaf oil, the mass of the adhered biofilm was scraped using a sterile cell scraper and thoroughly homogenised in 100 µL of sterile normal saline and then the suspension was serially diluted (10^-1^ to 10^-6^). Subsequently, an aliquot of 100 µL was plated on SDA medium. Agar plates were incubated at 37ºC for 24 h. After incubation, the colony forming units were counted and the results were expressed in CFU/mL.^29^

*Scanning electron microscopy (SEM) of forming biofilms in the presence of C. verum leaf oil* - Ultra structural properties of biofilms formed with the presence of different concentrations of *C. verum* oil was evaluated using SEM.^24^

Sterile cover slips were placed in 12-well cell culture cluster separately. Coverslips were immersed in 1 mL standardised cell suspension (1 x 10^7^ cells/mL) to ensure uniform *Candida* adhesion and incubated for 120 min at 37ºC in a shaker incubator (Nova Instruments, Brazil). Then the cover slips were moved carefully using a sterile forceps and gently immersed in new 12-well plate containing 0.5 mg/mL, 1.0 mg/mL and 2 mg/mL *C. verum* oil in RPMI 1640 (1 mL/well). Coverslips were incubated aerobically for 24 h at 37ºC. After 24 h incubation, the cover slips with formed biofilms in the presence of EO dilutions were subsequently washed three times with sterile PBS. Then they were transferred to a new 24-well cell culture cluster containing 2.5% glutaraldehyde at 4ºC. After fixing with glutraldehyde for 60 min, samples were dehydrated in a series of ethanol solutions (70% for 10 min, 80% for 10 min, 95% for 10 min and 10 min in absolute ethanol), and air-dried overnight in an incubator prior to sputter coating with gold. Chlorhexidine digluconate (0.25 mg/mL and 0.125 mg/mL) and Fluconazole (0.008 mg/mL) were used as positive controls to compare the post-exposure biofilm architecture with common antifungals and *C. verum* leaf oil treatment.

*Biofilm progression analysis (Time Lapse Microscopy)* - Progression of *Candida in-vitro* biofilms in the presence of *C. verum* leaf oil was evaluated using Time Lapse Microscopy. For analysis of biofilm progression in the presence of *C. verum* leaf oil, *C. albicans* (ATCC MYA-2876); *C. dubliniensis* (ATCC MYA-646) and *C. tropicalis* (ATCC 750) were adhered on to the polystyrene surface of 24-well cell culture cluster and treated with three concentrations (0.5 mg/mL, 1.0 mg/mL and 2.0 mg/mL) of *C. verum* leaf oil as explained previously. During the 24 h incubation at 37ºC after adding the treatment, images of forming biofilms were captured at time 0, 4 h, 8 h, 12 h, 16 h, 20 h and 24 h using time-lapse microscope^24^ (ZEISS ApoTome.2).

*In-vivo toxicity of C. verum essential oil on G. mellonella larvae* - The *in-vivo* toxicity experiment was performed on *G. mellonella* larvae as described previously by Wijesinghe (2019).^24^ Study groups and control group (n = 10) of larvae (RJ Mous Livebait, The Netherlands) were selected and selected larvae were kept in a Petri dishes (10/petri dish). Larvae with colour changes in their bodies including dark pigmentations or apparent melanisation, and larvae outside the body weight of 0.2- 0.3 g were excluded. Dilutions of EO of *C. verum* leaf were prepared in sterile PBS (Concentrations ranging from 0.5 to 1000 mg/mL).

Ten (10) µL of EO dilutions were injected in to the haemocele through the last left pro-leg of larvae using a 1 mL syringe. The prick area was decontaminated with 70% ethanol prior to administration of EO. Larvae containing dishes were then covered with a lid and kept in an aerobic incubator at 37ºC. 10 µL Sterile PBS with 0.05% Tween 80 was administered to negative control group (n = 10).

Viability of larvae was monitored by visual inspection of the body appearance (brown-dark brown colour) and by lack of body movement. The experiment was repeated two times.

*Statistical analysis* - The statistical analysis was carried out by using the software Statistical Package for Social Sciences (SPSS) version 16. Multiple means of more than three data sets were compared using one way analysis of variance (ANOVA) and two way ANOVA. The level of significance was taken at 5% (p < 0.05).

## RESULTS

*Chemical composition* - Identified and characterised chemical constituents and their relative abundance were represented in Table I. The most abundant active compound was Eugenol (77.22%). Benzyl benzoate (4.53%), Trans caryophyllene (3.39%), Acetyle eugenol (2.75%) and Linalool (2.11%) were found as minor components with a > 95% similarity between the spectra (NIST) (Table I).

**TABLE I:** Chemical constituents and relative abundunce of *Cinnamomum verum* leaf essential oil (EO) detected by gas chromatography-mass spectrometry (GC-MS)

Retention time (min)	Compound	% Abundance
21.41	Eugenol	77.22
36.65	Benzyl Benzoate	4.53
23.67	Trans caryophyllene	3.39
28.03	Acetyl eugenol	2.75
10.56	Linalool	2.11
17.45	Trans-Cinnamaldehyde	1.69
24.71	Acetic acid cinnamyl ester	1.49

*MIC* - Table II represents the MIC values corresponding to the *C. verum* EO and the positive control chlorhexidine digluconate. Both *C. verum* and chlorhexidine digluconate exhibited a similar efficacy on planktonic *C. albicans* (ATCC MYA-2876) and *C. dubliniensis* (ATCC MYA-646). *C tropicalis* (ATCC 750) was more susceptible for chlorhexidine digluconate compared to *C. verum* leaf oil.

**TABLE II:** Minimum inhibitory concentration (MIC) of *Candida* spp. Experiment was made in triplicates with three individual experiments. Chlorhexidine digluconate was used as positive control

Organism		*Cinnamomum verum* oil mg/mL	Chlorhexidine digluconate mg/mL
1	*C. albicans* (ATCC 5314)	1.0	1.0
2	*C. tropicalis* (ATCC 750)	1.0	0.5
3	*C. dubliniensis* (ATCC MYA-646)	1.0	1.0

**Fig. 1:**
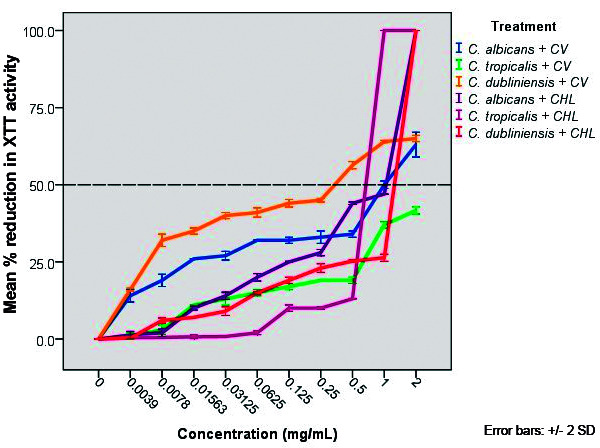
percentage reduction in XTT metabolic activity of *Candida albicans* (ATCC MYA-2876), *C. tropicalis* (ATCC 750) and *C. dubliniensis* (ATCC MYA-646) in the presence of different concentrations of *Cinnamomum verum* (CV) leaf oil and chlorhexidine digluconate (CHL). All error bars represent the ± 2 standard deviations (SD).

*Effect on initial adhesion* - The results of the microtiter plate XTT method in order to determine the effect of *C. verum* leaf oil on *Candida* adhesion on to a polystyrene surfaces was showed in Fig. 1.

According to obtained data, *C. verum* leaf oil effectively reduce the adhesion of all test strains on to polystyrene surface compared to negative control. *C. albicans* achieved 50% reduction in adhesion with 1.0 mg/mL concentration, whereas, *C. tropicalis* and *C. dubliniensis* showed 50% reduction in adhesion with > 2.0 mg/mL and 0.34 mg/mL concentrations of oil respectively. Chlorhexidine digluconate exhibited 50% reduction in adhesion of *C. albicans*, *C. tropicalis* and *C. dubliniensis* with the concentrations of 1.06 mg/mL, 0.72 mg/mL and 1.32 mg/mL respectively (Table III).

**TABLE III:** Minimum concentrations of *Cinnamomum verum* leaf essential oil (EO) and chlorhexidine digluconate (CHL) required to reduce the adhesion of *Candida albicans* (ATCC MYA-2876), *C. tropicalis* (ATCC 750) and *C. dubliniensis* (ATCC MYA-646) by 50%

	*C. albicans*		*C. tropicalis*		*C. dubliniensis*	
	*C. verum*	CHL	*C. verum*	CHL	*C. verum*	CHL
Concentration (mg/mL)	1.0	1.06	> 2.0	0.72	0.2	1.32

**Fig. 2:**
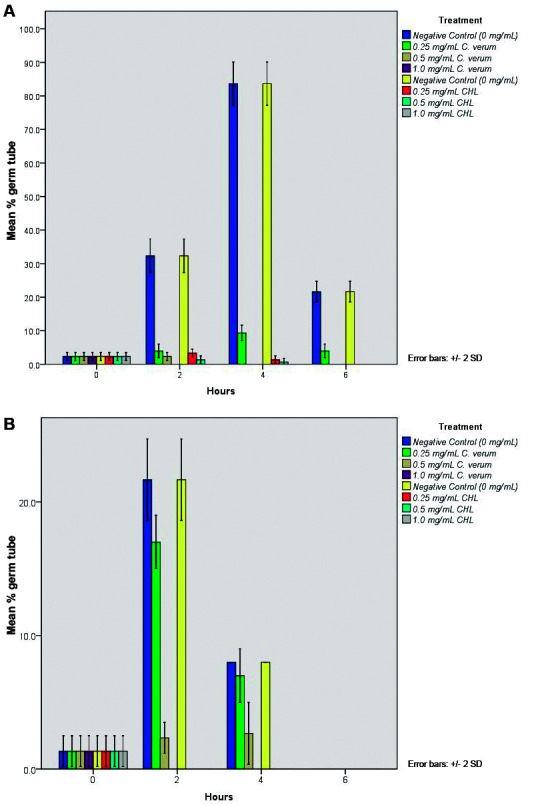
percentage germ tube formation of (A) *Candida albicans* (ATCC MYA-2876) and (B) *C. dubliniensis* (ATCC MYA-646) with 0.25, 0.5 and 1.0 mg/mL *Cinnamomum verum* leaf oil and chlorhexidine digluconate within 6 h test period with 2 h intervals. 0 mg/mL concentration indicates the negative control. All error bars represent the ± 2 standard deviations (SD).

*Effect of C. verum leaf oil on germ tube formation* - Fig. 2 shows the percentage of germ tube forming cells in the presence of 0.25, 0.5 and 1.0 mg/mL of *C. verum* leaf oil and chlorhexidine digluconate throughout 6 h experiment period.

According to obtained data, both *C. verum* leaf oil and chlorhexidine digluconate significantly reduced the germ tube formation of *C. albicans* and *C. dubliniensis* (p < 0.05) with all tested concentrations. Both *C. verum* leaf oil and chlorhexidine digluconate at 1.0 mg/mL (MIC) concentration completely inhibited the germ tube formation of both *Candida* species throughout the experiment period (6 h).

**Fig. 3:**
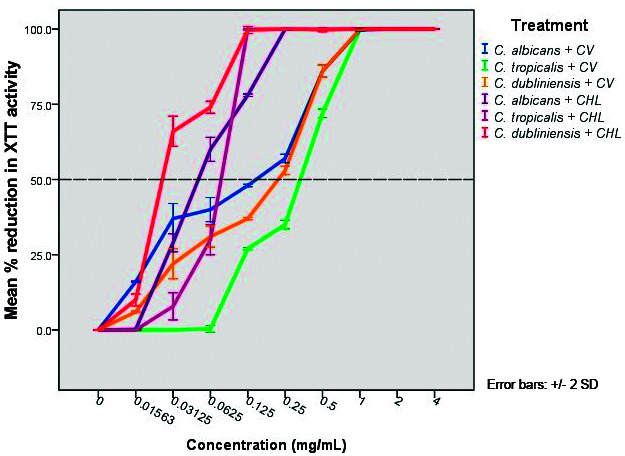
percentage reduction in XTT metabolic activity of *Candida albicans* (ATCC MYA-2876), *C. tropicalis* (ATCC 750) and *C. dubliniensis* (ATCC MYA-646) forming biofilms with the presence of different concentrations of *Cinnamomum verum* (CV) leaf oil and chlorhexidine digluconate (CHL). All error bars represent the ± 2 standard deviations (SD). All error bars represent the ± 2 standard deviations (SD).

*Minimum biofilm inhibitory concentration of forming biofilms* - In this experiment, minimum concentration of *C. verum* leaf oil required to inhibit the biofilm formation by 50% (compared to negative control) was determined. Biomass/viability of formed biofilms in the presence of essential oil was determined using XTT viability assay. Fig. 3 showed the percentage reduction of XTT metabolic activity of forming biofilms compared to negative control.

According to obtained data, concentrations required to reduce the biofilm formation by 50% as follows (Table IV).

**TABLE IV:** Minimum biofilm inhibitory concentrations (MBIC_50_) for forming biofilms of *Candida albicans* (ATCC MYA-2876), *C. tropicalis* (ATCC 750) and *C. dubliniensis* (ATCC MYA-646)

	*C. albicans*		*C. tropicalis*		*C. dubliniensis*	
	*Cinnamomum verum*	CHL	*C. verum*	CHL	*C. verum*	CHL
MBIC_50_ (mg/mL)	0.15	0.05	0.35	0.08	0.2	0.025

CHL: chlorhexidine digluconate.

**Fig. 4:**
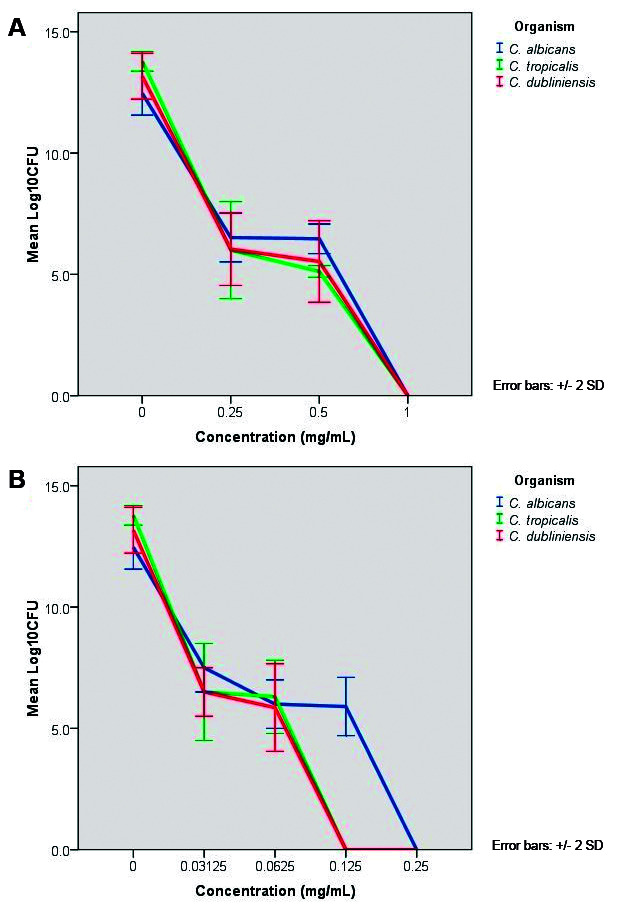
Log colony forming unit (CFU) values of forming *Candida albicans* (CA) (ATCC MYA-2876), *C. tropicalis* (CT) (ATCC 750) and *C. dubliniensis* (CD) (ATCC MYA-646) biofilms in the presence of different concentrations of (A) *Cinnamomum verum* (CV) leaf oil and (B) chlorhexidine digluconate (CHL). All error bars represent the ± 2 standard deviations (SD).

*MTC* - Minimum concentration of *C. verum* leaf oil required to prevent the biofilm formation completely by killing *Candida* cells was determined using CFU assay. Fig. 4 represents the viability of forming biofilms with the presence of different concentrations of *C. verum* leaf oil and chlorhexidine digluconate determined by CFU assay on forming biofilms. Table V represents the minimum concentration of treatments required to prevent the biofilm formation of test strains completely.

1.0 mg/mL *C. verum* kill the forming biofilms of all test strains, *C. albicans* (ATCC MYA-2876), *C. tropicalis* (ATCC 750) and *C. dubliniensis* (ATCC MYA-646). Chlorhexidine digluconate kills forming biofilms of *Candida* species with low concentrations compared to *C. verum* leaf oil. For *C. albicans*, the killing concentration with chlorhexidine digluconate was 0.25 mg/mL (Four times low concentration compared to that value of *C. verum* leaf oil) and for *C. tropicalis* and *C. dubliniensis*, killing concentration was 0.125 mg/mL (Eight times less concentration compared to that value of *C. verum*).

**TABLE V:** Concentrations of *Cinnamomum verum* leaf oil and chlorhexidine digluconate that kill the forming biofilms (MTC) of *Candida albicans* (ATCC MYA-2876), *C. tropicalis* (ATCC 750) and *C. dubliniensis* (ATCC MYA-646) completely

Test strain	Concentration requires to kill the forming biofilms (mg/mL)	
	*C. verum* oil	Chlorhexidine digluconate
*C. albicans* (ATCC MYA-2876)	1.0	0.25
*C. tropicalis* (ATCC 750)	1.0	0.125
*C. dubliniensis* (ATCC MYA-646)	1.0	0.125

**Fig. 5:**
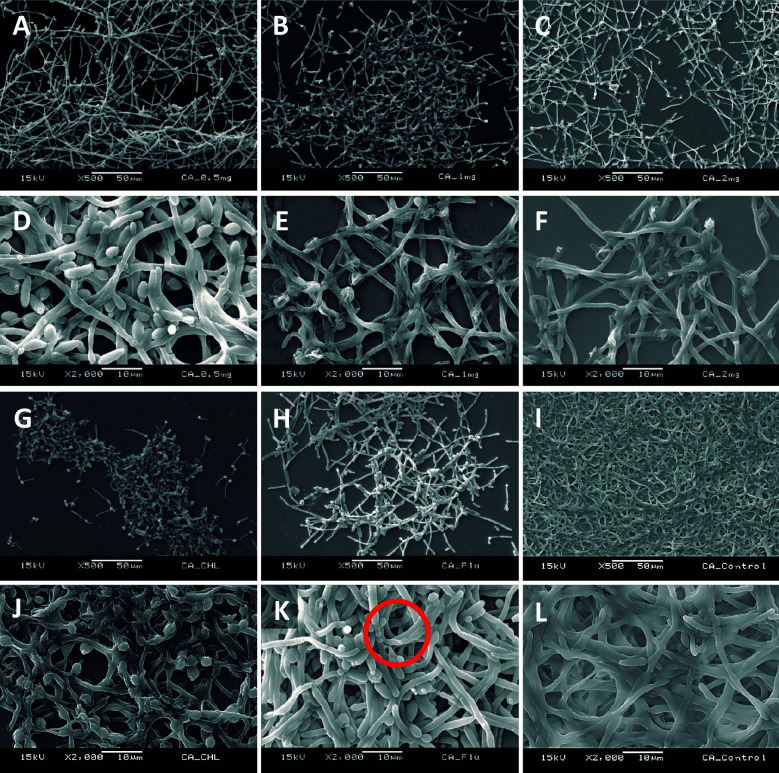
scanning electron microscopy (SEM) images of *Candida albicans* (ATCC MYA-2876) forming biofilms in the presence of 0.5 mg/mL *Cinnamomum verum* leaf oil (A and D), 1.0 mg/mL *C. verum* leaf oil (B and E), 2 mg/mL *C. verum* leaf oil (C and F), 0.25 mg/mL chlorhexidine digluconate (G and J) and 0.008 mg/mL Fluconazole (H and K). I and L: negative control. Red circles: cell wall deformities with treatments.

**Fig. 6:**
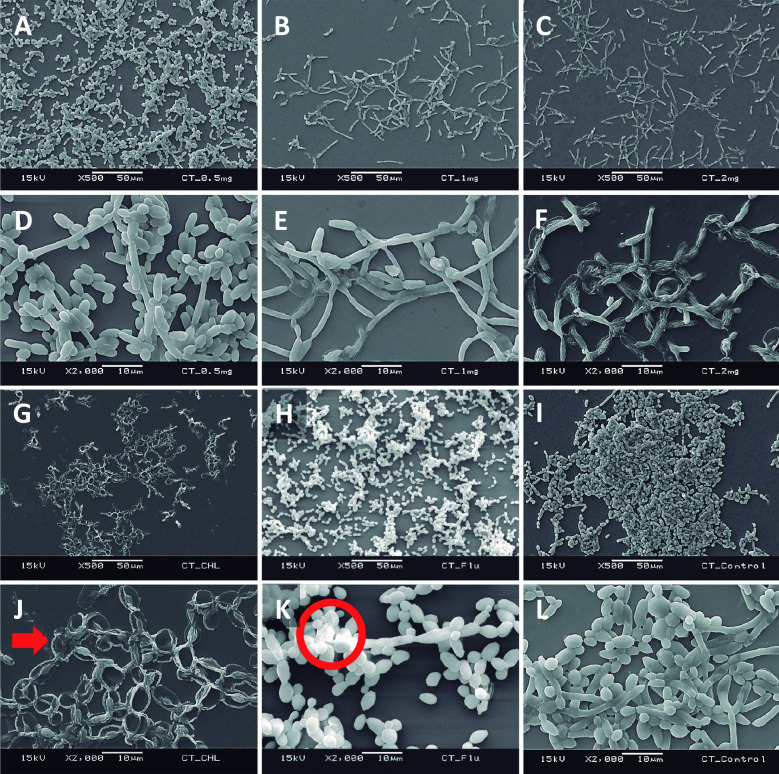
scanning electron microscopy (SEM) images of *Candida tropicalis* (ATCC 750) forming biofilms in the presence of 0.5 mg/mL *Cinnamomum verum* leaf oil (A and D), 1.0 mg/mL *C. verum* leaf oil (B and E), 2.0 mg/mL *C. verum* leaf oil (C and F), 0.125 mg/mL chlorhexidine digluconate (G and J) and 0.008 mg/mL Fluconazole (H and K) I and L: negative control. Red circle: cell wall deformities with treatments. Red solid arrow: leakages of intracellular components.

**Fig. 7:**
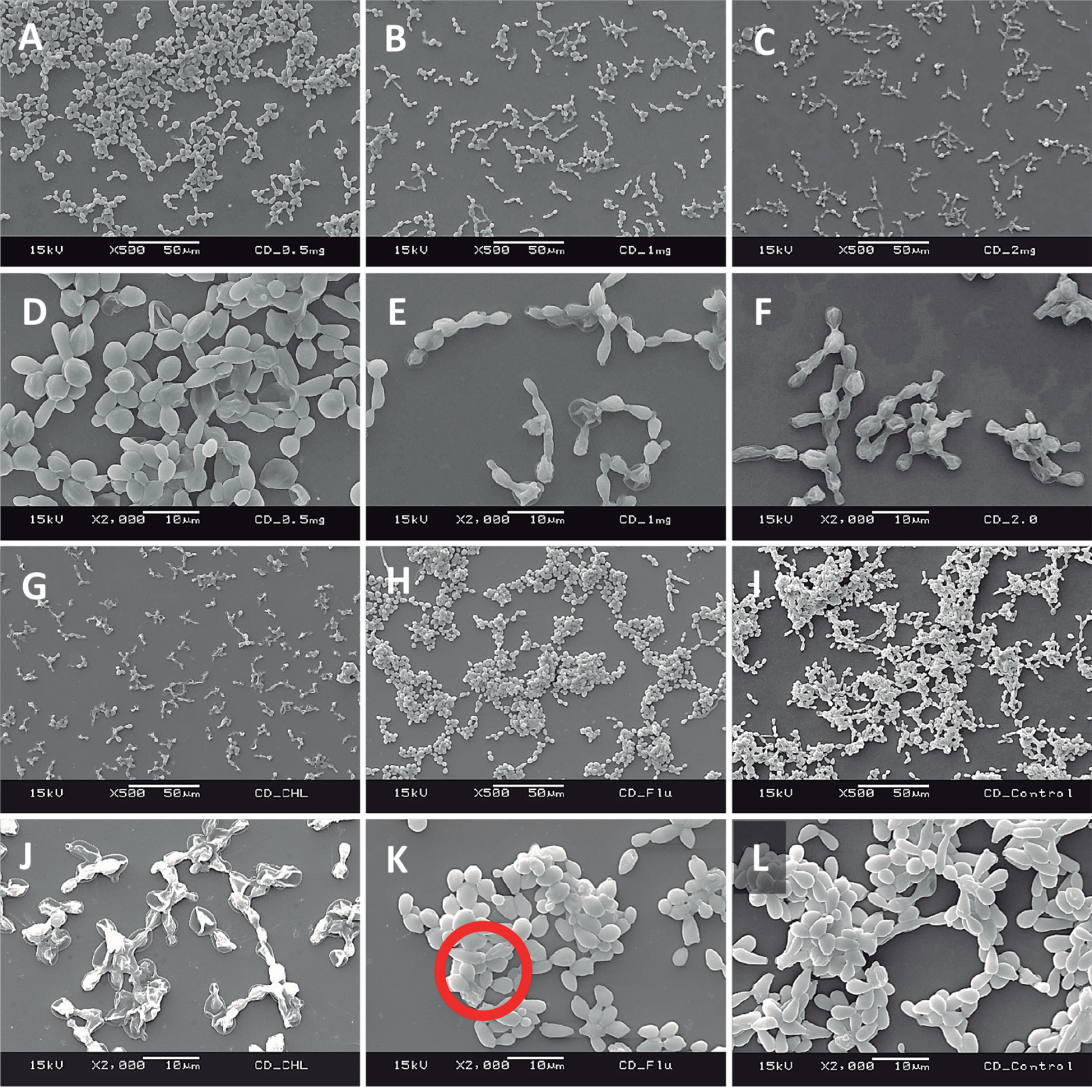
scanning electron microscopy (SEM) images of *Candida dubliniensis* (ATCC MYA-646) forming biofilms in the presence of 0.5 mg/mL *Cinnamomum verum* leaf oil (A and D), 1.0 mg/mL *C. verum* leaf oil (B and E), 2mg/mL *C. verum* leaf oil (C and F), 0.125 mg/mL chlorhexidine digluconate (G and J) and 0.008 mg/mL Fluconazole (H and K) I and L: negative control. Red circle: cell wall deformities with treatments.

*SEM of forming biofilms* - Ultrastructure of forming biofilms of *C. albicans* (ATCC MYA-2876), *C. tropicalis* (ATCC 750) and *C. dubliniensis* (ATCC MYA-646) in the presence of 0.5, 1.0 and 2.0 mg/mL *C. verum* leaf oil, chlorhexidine digluconate and Fluconazole was qualitatively evaluated by SEM (Figs 5, 6, 7).

*Cinnamomum verum* leaf oil caused the *Candida* cell shrinkage by damaging the cell wall and cause leakage of intracellular materials. These effects are concentration dependent. Maximum cell damage was observed with 2 mg/mL *C. verum* leaf oil. And *C. verum* leaf oil reduced the biofilm development and biofilm cell density.

**Fig. 8:**
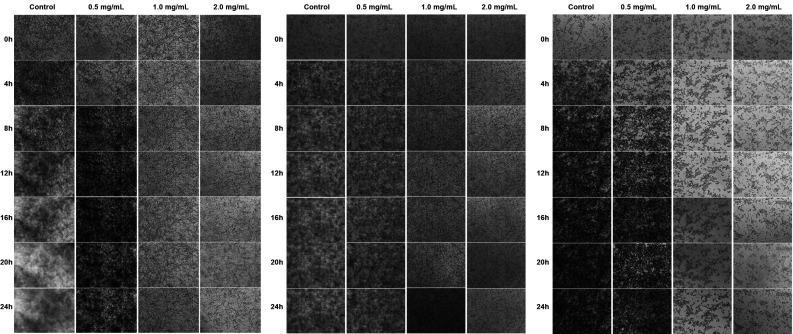
(A) Time Lapses images of developing biofilms of *Candida albicans* (ATCC MYA-2876). Each column represents the concentration of *Cinnamomum verum* leaf oil treatment. (B) Time Lapses images of developing biofilms of *C. tropicalis* (ATCC 750). Each column represents the concentration of *C. verum* leaf oil treatment. (C) Time Lapses images of developing biofilms of *C. dubliniensis* (ATCC MYA-646). Each column represents the concentration of *C. verum* leaf oil treatment.

*Biofilm progression analysis using time lapses microscope* - Fig. 8A-C indicate the Time lapses microscopic images of forming biofilms of *C. albicans* (ATCC MYA-2876); *C. dubliniensis* (ATCC MYA-646) and *C. tropicalis* (ATCC 750) without essential oil treatment, 0.5 mg/mL, 1.0 mg/mL and 2.0 mg/mL *C. verum* leaf oil treatment for 24 h time period.

The progression of the untreated (negative) control biofilm demonstrates excessive biofilm developement throughout the observation period. 0.5 mg/mL concentration of *C. verum* leaf caused retardation of biofilm development of *Candida* spp. 1.0 mg/mL and 2.0 mg/mL *C. verum* leaf oil completely inhibited *C. tropicalis* and *C. dubliniensis* cell proliferation and biofilm development from 0 h.

With 1.0 mg/mL *C. verum* leaf oil, *C. albicans* exhibited slight cell proliferation up to 8 h, while 2.0 mg/mL completely inhibited biofilm development.

**Fig. 9:**
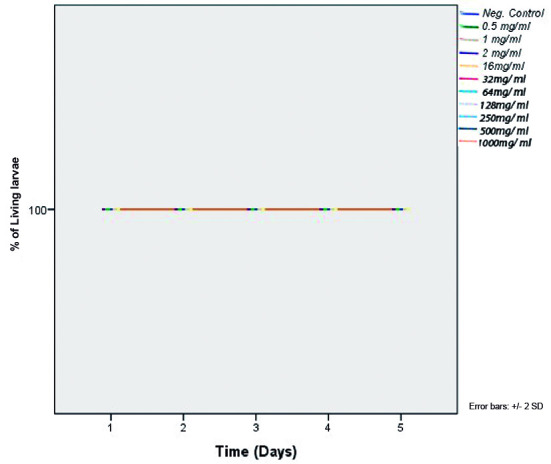
survival rate of *Galleria mellonella* larvae after administration of *Cinnamomum verum* leaf oil over five days experiment period. Control curve was obtained by administrating sterile phosphate buffered saline (PBS) in to larvae.

*In-vivo toxicity of C. verum leaf oil (G. mellonella larvae)* - *In-vivo* toxicity of *C. verum* leaf EO was evaluated by using *in-vivo G. mellonella* larvae model after treatment with different concentrations of EO. 100% survival of the treated larvae was observed at all concentrations tested (0.5, 1.0, 2.0, 16.0, 32.0, 64.0, 128.0, 250.0, 500.0, 1000.0 mg/mL) throughout whole experiment period (5 d) which indicates the non-toxicity of *C. verum* leaf EO on experimental model (Fig. 9).

## DISCUSSION

*Candida* spp. displays many virulent factors that contribute to successfully colonise in host tissues. Adhesion to host surfaces (which is the initial step of biofilm formation), germ tube formation, biofilm formation, secretion of proteinases and other hydrolytic enzymes and aldehyde production are some of them.^30^ Phytomedicinal therapeutics is becoming more popular in modern word because of the high occurrence of the antimicrobial resistance and increased virulence of microbial pathogens.^21^ On the other hand, easy access, lack of toxicity to the host, high efficacy and cost effectiveness contribute the widespread use of antimicrobial natural products as therapeutic alternatives.^21^ This study was conducted to evaluate the efficacy of such plant derived antimicrobial agent, *C. verum* leaf EO on controlling the virulence mechanisms of the most common human fungal pathogen, *Candida* spp. Further the toxicity on *in-vivo* laboratory experiment model, *G. melonella* larvae was also evaluated.

Adhesion to the host surfaces or abiotic surfaces is the very initial step of colonisation of the microbial pathogens. 1.0 mg/mL concentration of EO reduced adhesion of *C. albicans* by 50%, whereas, > 2.0 mg/mL and 0.34 mg/mL concentrations of oil reduced *C. tropicalis* and *C. dubliniensis* adhesion by 50% respectively. Reference antifungal chlorhexidine digluconate exhibited 50% of reduction in adhesion to *C. albicans*, *C. tropicalis* and *C. dubliniensis* with the concentrations of 1.06 mg/mL, 0.72 mg/mL and 1.32 mg/mL. Importantly, 50% reduction of adhesion of *C. albicans* and *C. dubliniensis* was achieved with lower concentrations of *C. verum* leaf oil compared to chlorhexidine digluconate control which indicates the higher efficacy of *C. verum* EO in anti-adhesion of those two strains. According to data obtained, 2.0 mg/mL chlorhexidine digluconate completely inhibit the adhesion of all test strains, but the observation may be due to killing of organisms completely during test period (2 h). Since adhesion on to the host surfaces is one of the major virulent factor and initial step of biofilm formation, the anti-adhesion properties of *C. verum* EO on *Candida* spp. contributes to its’ aptness as an antimicrobial agent.

Germ tube formation is another major virulent factor of *C. albicans* and *C. dubliniensis* that enables the absorption of nutrients from host tissues by invading them. Effect of *C. verum* at sub-MIC (0.25 and 0.5 mg/mL), both *C. verum* leaf oil and chlorhexidine digluconate significantly reduce the germ tube formation of both *C. albicans* and *C. dubliniensis* whereas MIC (1.0 mg/mL) completely inhibit the germ tube formation of test strains. With time (for *C. albicans*, after 2 h and for *C. dubliniensis*, after 4 h) the germ tube formation in all groups including control group gradually decreased since produced germ tubes transformed to pseudo hyphae and hyphae. There are no published scientific evidences available on effect of *C. verum* leaf EO on adhesion and germ tube formation of *Candida* spp. Current study was designed to find the efficacy of Cinnamon leaf oil on controlling virulent factors of *Candida* spp. in order to fill this gap of available data. Adhesion and germ tube formation are key attributes of infection causing ability of *Candida* and therefore antimicrobial substances which interfere with these factors are considered as potential anti-*Candida* agents. Hence *C. verum* leaf EO can be developed as a therapeutic alternative against *Candida* infections.

Biofilm is a surface attached microbial communities embedded in an extracellular matrix derived from cells and environment.^21^ Biofilms are more resistant to physical and chemical stresses compared to their planktonic counterpart as biofilms exhibit various mechanisms to neutralise those external stresses. Extracellular biofilm matrix acts as a physical or chemical barrier for diffusion of antimicrobial agents into the biofilm. Further, with the limited availability of nutrients, the biofilm community shifts towards slow or no growth status from exponential growth. All above factors and induction of a biofilm phenotype and quorum sensing contribute to high resistance to antimicrobial agents of a biofilm.^31^ ^,^ ^32^ Since the resistance of biofilms to available antimicrobial strategies is becoming more widespread, more studies should be carried out in order to invent novel, non-toxic, inexpensive and effective treatment options specially by controlling the virulence of the pathogen effectively. In the current study, efficacy of *C. verum* leaf EO as a potential phytomedicinal agent was evaluated on forming *Candida* biofilms. *C. verum* EO exhibited a potential antibiofilm effect on forming biofilms of *C. albicans* (ATCC MYA-2876), *C. tropicalis* (ATCC 750) and *C. dubliniensis* (ATCC MYA-646) test strains. Concentrations required to reduce the biofilm development by 50% (Compared to negative control) were 0.15, 0.35 and 0.2 mg/mL for three test strains respectively. And the MTC (concentrations of EO required to completely cease the biofilm development) was 1.0 mg/mL for all test strains. Importantly, these MTC values are lower than MIC and MFC values of test strains which indicates the high potency of killing of forming biofilms of *C. verum* EO though biofilms are considered as more resistant microbial communities than its planktonic counterpart. These data suggest the potential action of *C. verum* oil on interrupting the normal process of formation of *Candida* biofilms.

Biofilm progression under the chemical stress of *C. verum* leaf oil was visualised with time lapses microscope and results obtained from this experiment confirm above observation. 2 × MTC (2 mg/mL) of *C. verum* EO completely inhibited biofilm development of all test strains from time 0 h whereas slight cellular proliferation and biofilm development was observed in progressing *C. albicans* with MTC (1 mg/mL) from 0h to 8 h. After 8 h, no visible biofilm development was noted in *C. albicans* developing biofilms. *C. tropicalis* and *C. dubliniensis* biofilm development was completely inhibited at 0 h by MTC. Sub-MTC (0.5 mg/mL) causes retardation of biofilm development of all tested *Candida* strains. These results indicate the possible effective use of *C. verum* leaf EO as a biofilm preventive strategy. Further, data from SEM and time lapses microscopy reveal that the effect of EO on the progression of *Candida* biofilm is dose dependent.

SEM images were taken to understand the structure of the *Candida* biofilms developed with the presence of *C. verum* leaf EO. All biofilms of test strains exhibited reduced cell density, cell wall damages, cell wall deformities and leakages of intracellular materials with treatment of cinnamon leaf oil. Importantly, SEM images confirm the dose dependent nature of effects of *C. verum* leaf essential oil. Fluconazole is the known antifungal agent which belongs to azole group and inhibits synthesis of fungal sterol, ergosterol by preventing the conversion of lanosterol to ergosterol.^33^ On the other hand chlorhexidine digluconate is a biguanide which is used as an antibacterial mouth rinse. It alters the morphology cells and damages the cell wall of microorganisms and releases intracellular components. It has been suggested as a well-known therapeutic antifungal agent for oral candidiasis.^34^ ^,^ ^35^ Since both antimicrobial agents have an effect on *Candida* cell wall, ultrastructure of forming *Candida* biofilms with 0.25 mg/mL chlorhexidine digluconate (for *C. albicans*), 0.125 mg/mL chlorhexidine digluconate (for *C. tropicalis* and *C. dubliniensis*) and 0.008 mg/mL Fluconazole (maximum recommended *in vitro* assay concentration, CLSI) were also visualised. Similar observations were obtained with the exposure to MTC of chlorhexidine digluconate and 0.008 mg/mL Fluconazole. The intensity of the post-exposure response of Fluconazole was minimal due to low concentration.

When considering chlorhexidine digluconate, it exhibited the similar effect as *C. verum* leaf oil (damaging cell walls of *Candida* cells and cause cytoplasmic leakages). Further, according to SEM observations, chlorhexidine digluconate decreased pseudo hyphae formation of *C. tropicalis* compared to negative control. Extracellular polymeric matrix that is characteristic of biofilms was not observed in any of the biofilms analysed using SEM since pre-treatments and electron beam of the equipment can cause the matrix destruction.

Though, the effect of *C. verum* oil on *Candida* biofilm formation is not well studied, few studies were conducted by various scientists to evaluate the efficacy of Cinnamon oil on virulence and biofilm depletion of different bacterial species. Kalia et al.^36^ in 2015 studied the anti-quorum sensing activity of cinnamon oil against *Pseudomonas aeruginosa* by measuring the inhibition of biofilm formation and other quorum sensing (QS) associated virulence factors such as proteolytic enzyme production and swarming activity and effective biofilm reduction and antagonist effect on QS. Further, Erfan and Marouf in 2019 observed a downregulation of virulence genes of respiratory bacterial pathogens (*Staphylococcus aureus*, *E. coli*, *Avibacterium paragallinarum* and *Pasteurella multocida* etc.) by cinnamon oil. They suggested the possible use of cinnamon oil for the control of antibiotic-resistant bacterial infections instead of the routine antibiotics.^37^ Another two studies conducted by Zhang et al. in 2020 and Kim et al. in 2014^38^ ^,^ ^39^ identified few anti-virulence mechanisms of *C. verum* bark oil including the inhibition of microbial toxins, interrupting the biofilm development and quorum sensing of pathogenic bacterial species. But the anti-virulence properties of *C. verum* leaf oil on fungal virulence was not widely studied.

Toxicological assessment of a natural product prior to its clinical application is an important aspect when the invention of a novel herbal therapeutic agent is concerned. The current study determines the *in-vivo* toxicity of *C. verum* EO using the *G. mellonella* experiment model. 100% survival was observed with *G. mellonella* larvae with all test concentrations ranging from 0.5 to 1000 mg/mL EO. This indicates the non-toxicity of *C. verum* leaf oil on experiment model. Since 1000 mg/mL is the neat concentration (undiluted) of *C. verum* leaf EO all dilutions including neat solution can be considered as non-toxic. Even though previous *in-vivo* studies conducted on animal models have also highlighted lack of significant toxicity with a significantly high therapeutic range of concentration of cinnamon bark oil,^22^ the *in-vivo* toxicity of cinnamon leaf oil on laboratory animal models was not well studied.

The results of the chemical analysis of the *C. verum* leaf EO agree with the findings of previous studies, which indicate that eugenol is its most abundant chemical component (77.22%) whereas the other chemical compounds appear in smaller concentrations.^40^

The high occurrence and mortality rates associated with Candidiasis emphasise the urgent necessity for the introduction of new therapeutic approaches to treat candidiasis. Various studies conducted by multiple research groups has provided important knowledge about the pathogenesis of candidiasis,^15^ ^,^ ^41^ and this knowledge should represent a starting point for the transformation of their findings into treatment strategies that can directly benefit patients suffering from *Candida* and other infections. More effective and earliest option among them is the invention of novel anti-virulence approaches for the control and treatment of *Candida* infections.^42^ Because germ tube formation, adhesion on to host surfaces and biofilm formation constitute the major virulence factors during candidiasis, they represent a clinically unexploited target for the innovation of such alternative anti-virulence strategies. In this study, felicity of a natural product, *C. verum* leaf EO as a non-toxic, anti-virulence strategy against *Candida* spp. was identified using both *in vitro* and *in vivo* experiment series.

Though a number of *in vitro* and *in-vivo* studies have used plant derived natural products for the treatment of *Candida* infections, the exact mechanism of action of the antifungal effect or the effect on Candidal physiology of those treatments were not clearly understood. The *C. verum* leaf EO used in the present study is a herbal product with potential for non-toxic therapeutic application in the treatment of candidiasis by interfering the virulence of *Candida*. However, further experiments including *in-vivo* toxicity investigations and clinical trials as well as molecular studies on gene regulation are recommended to develop an antifungal agent based on *C. verum* leaf EO.

In conclusion, this study provides evidence for the high efficacy of *C. verum* leaf EO as an anti-virulence approach against three key virulence factors of *Candida* spp., adhesion, germ tube formation and biofilm formation. The EO extracted from *C. verum* leaves has eugenol as its major active component and is non-toxic against *G. mellonella* larvae.
